# Efficacy and safety of empiric treatment with omeprazole continuous infusion in critically ill children with gastrointestinal bleeding

**DOI:** 10.3389/fped.2024.1270911

**Published:** 2024-04-08

**Authors:** Khalid W. Taher, Rahaf Yaseen, Mayas Alnan, Wejdan Aburas, Hala Khalil, Moath Alabdulsalam

**Affiliations:** ^1^ Pharmaceutical Care Division, King Faisal Specialist Hospital & Research Centre, Riyadh, Saudi Arabia; ^2^ College of Pharmacy, Alfaisal University, Riyadh, Saudi Arabia; ^3^ PGY1 Pharmacy Residency Program, Pharmaceutical Care Division, King Faisal Specialist Hospital & Research Centre, Riyadh, Saudi Arabia; ^4^ Therapeutic Affairs Deputyship, Ministry of Health, Riyadh, Saudi Arabia; ^5^ Department of Biostatistics, Epidemiology and Scientific Computing, King Faisal Specialist Hospital & Research Centre, Riyadh, Saudi Arabia; ^6^ Department of Pediatrics, King Faisal Specialist Hospital & Research Centre, Riyadh, Saudi Arabia

**Keywords:** omeprazole, continuous infusion, children, gastrointestinal bleeding, pediatric intensive care

## Abstract

**Introduction:**

Gastrointestinal bleeding (GI) is a prevalent condition among pediatric patients, with a reported incidence of 6.4%, often severe enough to require admission to the pediatric intensive care unit (PICU). There are multiple therapies utilized in the management of GI bleeding in pediatrics, among which continuous intravenous (IV) infusion of omeprazole is used off-label without standard pediatric dosing recommendations. Reviewing the current literature reveals a lack of studies assessing the efficacy, safety, and appropriate dosing regimen of continuous omeprazole infusion in children with GI bleeding. This study aimed to evaluate the efficacy and safety of continuous IV omeprazole infusion in comparison to other therapeutic modalities in children.

**Methods:**

This study is a single-center, retrospective chart review of children admitted to the PICU at King Faisal Specialist Hospital and Research Centre, Riyadh, Saudi Arabia. The treatment group included pediatric patients with GI bleeding and receiving omeprazole IV continuous infusion over ≥24 h while the control group included pediatric patients with GI bleeding managed using other therapies. Primary outcomes were the efficacy of omeprazole continuous infusion in stopping GI bleeding, and PICU length of stay (LOS). Secondary outcomes included instances of rebleeding post- therapy discontinuation, transfusion requirements, and the safety of omeprazole continuous infusion.

**Results:**

The study included 81 critically ill pediatric patients, 22 of whom received continuous infusion omeprazole while 59 received other therapies. The results indicated that patients in the control group had a significantly shorter PICU LOS (8 vs. 18.5 days, *p* < 0.001) and bleeding episode (4 vs. 10.5 days, *p* < 0.001) than those in the treatment group. However, no significant differences were observed between the two groups regarding secondary outcomes. The treatment group had a significantly lower all-cause mortality rate during hospitalization compared to the control group (16 patients [72.7%] vs. 56 patients [94.9%], respectively, *p* = 0.005).

**Conclusion:**

Empirical use of omeprazole continuous intravenous infusion in children with GI bleeding was not favorable in terms of shortening PICU LOS and duration of GI bleeding. Our study results provide evidence supporting the safety and tolerability of omeprazole continuous infusion. Additional larger studies are necessary to determine the implication of such results.

## Introduction

Gastrointestinal (GI) bleeding is a prevalent condition with a reported incidence of 6.4% in children, which can manifest in any part of the digestive system. Severe GI bleeding in children may lead to hemodynamic instability, necessitating admission to the pediatric intensive care unit (PICU). The management of GI bleeding in pediatric patients typically involves the use of acid suppression therapies, such as proton pump inhibitors (PPIs), vasoactive drugs, such as octreotide, and certain procedures, such as therapeutic endoscopy (1).

Pediatric patients require careful consideration when determining the pharmacokinetics and optimal dosage of intravenous omeprazole, particularly continuous infusion omeprazole. A study focused on children under 30 months old found that to maintain a gastric pH of over 4 for 24 h, a higher dosage of omeprazole (i.e., 40 mg/1.73 m^2^) was needed. The drug exhibited rapid elimination in plasma concentration vs. time curves. Although children at 2 years of age develops glucuronidation slower, oxidative capacity is thought to be greater compared to adults. These factors suggest the need for a higher and maintained dosing regimen (2). Intravenously administered proton pump inhibitors (PPIs) has been widely employed in acute gastrointestinal bleeding due to their efficacy. In this regard, the administration of a PPI bolus dose, followed by continuous infusion, may be deemed a viable therapeutic strategy (3). However, several significant adverse effects of PPIs have been reported, including hypersensitivity reactions, hypomagnesemia, interstitial nephritis, vitamin B-12 deficiency, and upper respiratory tract infections (4). A retrospective study was conducted to assess the incidence of hypersensitivity reactions to PPI, which revealed that out of 1,229 patients, only twelve had a history of PPI hypersensitivity reactions. Oral re-challenging was conducted on five patients using alternative anti-acid drugs, including PPIs, with none of them experienced any adverse reactions (5).

While continuous intravenous infusion of omeprazole has been reported for off-label use in the pediatric population, optimal dosage has yet to be established. A case study conducted in the United Kingdom showed the effectiveness of continuous intravenous infusion of omeprazole in managing upper gastrointestinal bleeding in a 3-month-old infant. The omeprazole infusion was initiated at a dose of 0.15 mg/kg/h and resulted in successful control of bleeding within 6 h. Notably, discontinuation of the infusion led to rebleeding, which was then managed by escalating the omeprazole dose to 0.3 mg/kg/h and concurrent surgical intervention, resulting in successful bleeding control (6).

A meta-analysis was conducted to assess the efficacy of intermittent omeprazole, compared to the continuous infusion regimen, pantoprazole 80 mg IV bolus followed by a continuous IV infusion 8 mg/h for 72 h, for the reduction of ulcer rebleeding in adult patients. The study concluded that intermittent omeprazole therapy was non-inferior to IV bolus plus continuous IV infusion of pantoprazole for the prevention of rebleeding within 7 days (7). A randomized prospective study was carried out in Romania to determine the efficacy of continuous IV infusion of esomeprazole vs. boluses and second-look endoscopy in preventing rebleeding in children with peptic ulcers who had already undergone primary therapeutic endoscopy. The study observed that high-dose continuous IV esomeprazole infusion (i.e., initial intravenous bolus of 1 mg/kg, followed by a continuous infusion of 0.1 mg/kg/h for 72 h) was as effective as second-look endoscopy and bolus esomeprazole in curbing ulcer rebleeding. In addition, the study showed that continuous esomeprazole infusion led to a reduction in children's discomfort and minimized the workload associated with a second endoscopy (8). In Saudi Arabia, a retrospective study was conducted on adult patients to compare the safety and efficacy of intermittent and continuous PPI infusions for non-variceal upper gastrointestinal bleeding. Results indicated no significant differences in rebleeding rate or mortality between the two administration methods, and both were well-tolerated and safe. Based on these findings, the study concluded that intermittent PPI infusion may be a cost-effective alternative to continuous infusion in managing non-variceal upper gastrointestinal bleeding (9).

Despite the clinical utility of continuous IV omeprazole infusion among pediatric patients, its effectiveness, safety, and optimal dosing with gastrointestinal bleeding remains under investigated. This study aims to assess the safety and efficacy of continuous intravenous omeprazole infusion in comparison to alternative therapies among critically ill pediatric population.

## Materials and methods

### Study design and population

This is a single-center retrospective cohort chart review study of children admitted to our 30-bed pediatric intensive care units (PICU) at King Faisal Specialist Hospital and Research Centre, Riyadh, Saudi Arabia from January 1, 2017, to September 30, 2022. The study subjects included children between one month and 14 years, who were admitted to the PICU with GI bleeding regardless of the reason for PICU admission. Exclusion criteria included patients who are <1 month old, >14 years old, those who received their therapy through a non-intravenous route, those who received prophylactic treatment with no evidence of GI bleeding and cases with incomplete medical records. The treatment group included pediatric patients with GI bleeding and receiving omeprazole IV continuous infusion, defined as any prescribed intravenous omeprazole order set to be infused over at least 24 h. The control group included pediatric patients with GI bleeding receiving other intravenous therapies, such as intermittent omeprazole dosing, Histamine (H2) receptor antagonists, and/or octreotide. In our study, GI bleeding was defined as the presence of fresh blood or melena anywhere in the GI tract, the appearance of blood or coffee ground aspirate in the nasogastric tube as documented by the attending physician or nurse, or evidence of GI bleeding confirmed by imaging. The institutional review board approved the study and a waiver of consent was obtained.

### Data collection

Data collection was carried out through the patient's electronic health record. Data collected included demographic details [age, gender, height, weight, body mass index (BMI)], clinical characteristics (vital signs, reason for PICU admission, comorbidities), as well as omeprazole dose, duration, and route of administration, and intravenous treatments used for GI bleeding (omeprazole, octreotide, and H2 receptor antagonists). Other collected data encompassed PICU length of stay, start, and end of GI bleeding, and rebleeding episodes following discontinuation of therapy, coagulation profile, blood transfusion requirement, electrolytes, and serum chemistry. Additionally, for the treatment and control group, data on hypersensitivity reactions and enteric infections were collected. As this study is a retrospective in nature, the selection of treatment options was based on the individual physician's judgement and preference at the time of patient care, and patients may have been initiated on ≥1 therapy based on their clinical status. Obesity status was determined based on the Centers for Disease Control and Prevention calculator for children ≥2 years of age that calculated BMI for age and sex (10). The World Health Organization criterion was used for defining obesity in children <2 years of age (11). Children ≥2 years of age were classified as obese if BMI was ≥95th percentile, while children <2 years of age were classified as obese if weight-for-length was ≥97.7th percentile.

### Study outcomes

The primary outcomes included the efficacy of continuous omeprazole infusion in stopping GI bleeding based on the physician documentation and the PICU length of stay (LOS). Secondary outcomes included rebleeding, which was defined as the presence of fresh blood or melena anywhere in the GI tract or the appearance of blood or coffee ground aspirate in the nasogastric tube, or evidence of GI bleeding confirmed by imaging within 48 h from the resolution of the previous bleeding episode, transfusion requirements, and the safety of continuous omeprazole infusion in the treatment group (electrolyte abnormalities, hypersensitivity reaction, and enteric infections).

### Statistical analysis

The data were analyzed using both descriptive and inferential statistics. The Chi-square test was used to compare categorical variables, and results presented as frequencies and percentages. The *t*-test or Mann–Whitney *U*-test was performed to compare continuous data, contingent on the normality of data distribution and reported as mean, standard deviation (SD) or median, interquartile range (IQR). For the sample size, the study included all patients who received omeprazole or alternative therapies. Therefore, the sample size was imposed based on the number of eligible patients during the prespecified time period. Statistical analyses were conducted with SPSS for Windows, Version 20 (SPSS Inc., Chicago), with a significant level set at 0.05.

## Results

### Patients' characteristics

**Figure 1:**
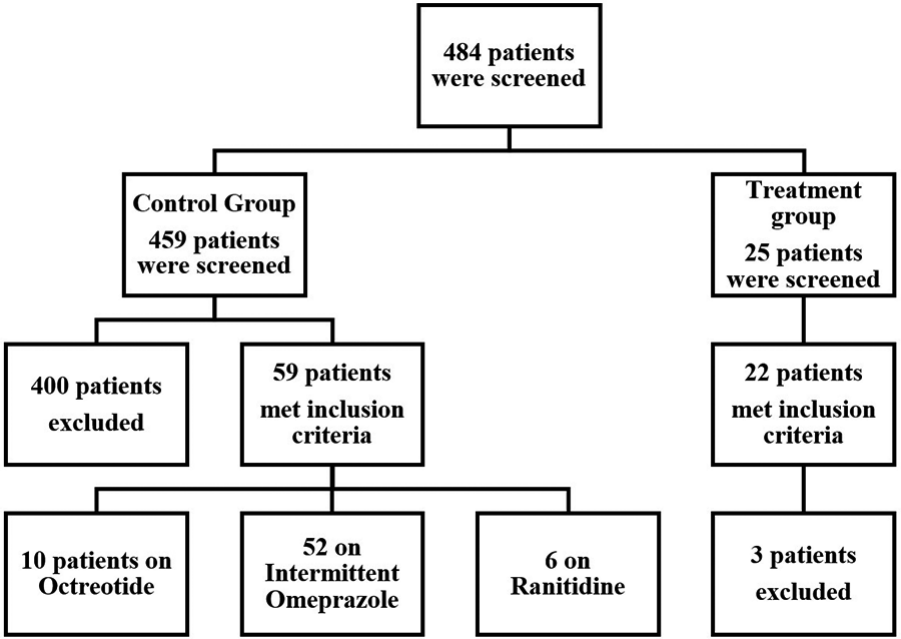
Flow diagram of patient screening and inclusion.

From an initial pool of 484 evaluated, 22 patients were included in the treatment group and 59 patients in the control group after applying exclusion criteria (Figure 1). Baseline characteristics of the included patients are presented in Table 1. The mean age in the treatment group was 7.9 years, with 63.6% males compared to a mean age of 5.8 years in the control group with 55.9% males (Table 1). The control group had a total of five patients (8.5%) who were classified as obese based on the BMI percentile while, no patients in the treatment group met the criteria for obesity. The incidence of both upper and lower GI bleeding was higher in the treatment group (14 patients, 63.7%) than in the control group, (20 patients, 33.9%, *p* = 0.054). In the control group, higher rates of upper GI bleeding were noted (33 patients, 55.9%) compared to the treatment group (7 patients; 31.8% although this difference was borderline significant, *p* = 0.054). Endoscopy was not performed for the majority of patients, reported in only 4 patients in the treatment group and 2 patients in the control group. Additionally, the reasons for GI bleeding varied between the two groups but were overall balanced. The most common reason was Graft-Versus-Host disease (GVHD), higher in the control group (16 patients, 27.1%) than in the treatment group, (4 patients, 18.2%, *p* = 0.407). Regarding comorbidities, the control group had a higher incidence of acute kidney and liver injury, while the treatment group had a higher incidence of coagulopathies and hypertension. The study groups were predominantly homogeneous and comparable in most aspects. A comprehensive analysis of the physiological and laboratory parameters recorded before and after intervention for both the control and treatment groups are presented in Table 2.

**Table 1:** Patient characteristics.

Variables	Treatment group (*n* = 22)	Control group (*n* = 59)	*p*-value
Age at ICU admission (years; mean ± SD)	7.9 ± 4.3	5.8 ± 4.2	0.055
BMI (kg/m^2^; mean ± SD)	14.8 ± 3.3	15.5 ± 3.3	0.35
Weight (kg), median (IQR)	19.4 (13.4)	15 (12)	0.083
Sex, Male (*n*, %)	14 (55.9)	33 (63.6)	0.53
Type of GI bleeding (*n*, %)			
Upper GI bleeding	7 (31.8)	33 (55.9)	0.054^a^
Lower GI bleeding	1 (4.5)	6 (10.2)	
Upper & lower GI bleeding	14 (63.7)	20 (33.9)	
Reason for GI bleeding (*n*, %)			
Neoplasm/mass	1 (4.6)	5 (8.5)	0.548
Ulcerative/erosive causes	3 (13.6)	6 (10.2)	0.659
Varices/liver cirrhosis	3 (13.6)	2 (3.4)	0.088
Thrombocytopenia without confirmed cause	2 (9.1)	3 (5.1)	0.505
GVHD	4 (18.2)	16 (27.1)	0.407
Others^b^	5 (22.7)	2 (3.4)	0.006
Unknown	4 (18.2)	25 (42.4)	0.043
Presence of coagulopathies^c^, Yes (*n*, %)	13 (59.1)	30 (50.8)	0.51
Presence of liver cirrhosis, Yes (*n*, %)	2 (9.1)	4 (6.8)	0.66^a^
Presence of acute liver injuries^c^, Yes (*n*, %)	2 (9.1)	27 (45.8)	<0.001^a^
Presence of chronic kidney injury, Yes (*n*, %)	2 (9.1)	5 (8.5)	1^a^
Presence of acute kidney injury^c^, Yes (*n*, %)	13 (59.1)	44 (74.6)	0.18
HTN, Yes (*n*, %)	10 (45.5)	13 (22)	0.038
Diabetes, Yes (*n*, %)	1 (4.5)	0 (0)	0.09^a^

BMI, body mass index; GI, gastrointestinal; HTN, hypertension; ICU, intensive care unit; SD, standard deviation; GVHD; graft-versus-host-disease.

*p*-values are obtained from chi-square test for categorical variables and from independent *t*-test for continuous variables.

^a^ *p*-values are obtained from Fisher's exact test.

^b^ Other causes included, febrile neutropenia, mucositis, viral infection, coagulopathy, and enterocolitis.

^c^ The acute disease state was assessed both at baseline and during the administration of the intervention.

**Table 2:** Pre- and post-intervention laboratory and physiological parameters.

Variables	Treatment group (*n* = 22)	Control group (*n* = 59)	*p*-value
Hemoglobin levels at start (g/dl)^b^	8.4 ± 2.1	8.8 ± 2.2	0.49^a^
Hemoglobin levels at end (g/dl)^b^	9.6 ± 1.5	8.2 ± 1.9	0.11^a^
Serum creatinine levels at start (mmol/L)	52.5 (59.0)	41.0 (99.0)	0.76
Serum creatinine levels at end (mmol/L)	50.0 (50)	78.0 (53.0)	0.11
Blood urea nitrogen levels at start (mmol/L)	14.9 (15.0)	8.5 (9.0)	<0.001
Blood urea nitrogen levels at end (mmol/L)	13.5 (12.3)	10.8 (20.2)	0.4
Platelet counts at start (×10^3^/ml)	57.5 (61.0)	28.0 (43.0)	0.08
Platelet counts at end (×10^3^/ml)	41.0 (52.0)	30.0 (36.0)	0.59
Systolic BP at start (mmHg)	111 (18.4)	101.4 (15.5)	0.053
Diastolic BP at start (mmHg)	62.6 (15.3)	59.9 (12.6)	0.633
Systolic BP at end (mmHg)	97.8 (26.3)	90.5 (28.0)	0.288
Diastolic BP at end (mmHg)	57.0 (19.0)	49.0 (18.8)	0.099
Heart rate at start (bpm)	119.7 (16.2)	131.1 (22.3)	0.013
Heart rate at end (bpm)	100.5 (35.6)	115.1 (30.6)	0.077
Respiratory rate at start (breaths/min)	29.7 (12.0)	33.0 (13.3)	0.286
Respiratory rate at end (breaths/min)	33.1 (12.8)	27.2 (13.0)	0.073

BP, blood pressure; bpm, beats per min.

*p*-values are obtained from Mann–Whitney *U*-test, except were indicated otherwise.

^a^ *p*-value from independent *t*-test.

^b^ Values are presented as mean ± SD. All other values are presented as median (interquartile range).

### Primary and secondary outcomes

**Figure 2:**
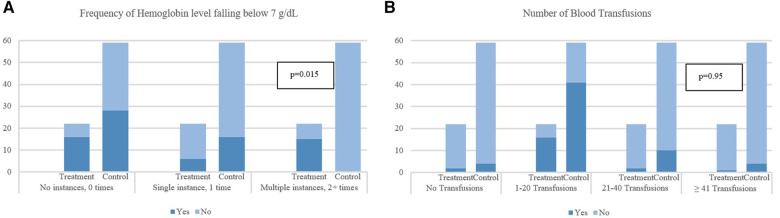
Impact of gastrointestinal bleeding on hemoglobin levels and frequency of blood transfusions. (**A**) Frequency of Hemoglobin level falling below 7 g/dl. (**B**) Number of Blood Transfusions. *p*-values are obtained from chi-square test or Fisher's exact test for categorical variables and from Mann–Whitney *U*-test for continuous variables. *p-values are obtained from Fisher's exact test.

As shown in Table 3, a significant difference was observed in the median (IQR) PICU LOS with the treatment group recording 18.5 (13.8–36.5) days vs. the control group's 8 (3–20) days (*p* < 0.001). Furthermore, the duration of the bleeding episode was significantly shorter in patients in the control group compared to the treatment group (4 [IQR: 1–7] days vs. 10.5 [IQR: 6.0–19.3] days, *p* < 0.001). Rebleeding incidence was slightly higher in the control group [7 patients (11.8%)] compared to the treatment group [2 patients (9.1%)], but not statistically significant, *p* = 0.724. Concerning safety, there were no recorded instances of hypersensitivity reactions in either group. The control group had one enteric infection case, whereas the treatment group had none. Additionally, the incidence of nausea and vomiting seemed higher in the control group [15 patients (25.4%)] compared to the treatment group [2 patients (9.1%)], however, this difference was not statistically significant, *p* = 0.108. The incidence of hypomagnesemia was higher in the control group [44 patients (74.6%)] compared to the treatment group [13 patients (59.1%)], though not statistically significant, *p* = 0.175. Table 4 shows the detailed therapeutic intervention data for both treatment and control groups. In the treatment group, the use of continuous IV omeprazole infusion exhibited significantly fewer instances of hemoglobin levels falling below 7 g/dl compared to the control group (no instances in 16 patients [72.7%] vs. 28 patients [47.5%] respectively, *p* = 0.015). Similarly, a single instance was found in 6 patients [27.3%] in the treatment group vs. 16 patients [27.1%] in the control group, *p* = 0.015. Although, multiple instances of hemoglobin dropping below the threshold occurred in the treatment group [15 patients (25.4%)], no multiple instances were found in the control group. Additionally, fewer instances of multiple blood transfusions occurred in the treatment group in comparison to the control (no transfusions in 2 patients [9.1%] vs. 4 patients [6.8%], 1–20 transfusions in 16 patients [72.7%] vs. 41 patients [69.5%], 21–40 transfusions in 3 patients [13.6%] vs. 10 patients [16.9%], ≥41 transfusions in 1 patient [4.5%] vs. 4 patients [6.8%], *p* = 0.95) respectively, all was not statistically significant (Figure 2).

**Table 3:** Comparison of primary and secondary outcomes between treatment and control groups.

Outcomes	Treatment group (*n* = 22)	Control group (*n* = 59)	*p*-value
Primary outcomes			
PICU LOS [days; median (IQR)]	18.5 (13.8–36.5)	8 (3–20)	<0.001
Length of bleeding [days; median (IQR)]	10.5 (6–19.3)	4 (1–7)	<0.001
Secondary outcomes			
Rebleeding after discontinuation, Yes (*n*, %)	2 (9.1)	7 (11.9)	0.724
Hypersensitivity reactions, Yes (*n*, %)	0 (0)	0 (0)	NA
Enteric infections, Yes (*n*, %)	0 (0)	1 (1.7)	0.54
Nausea and vomiting, Yes (*n*, %)	2 (9.1)	15 (25.4)	0.14
Hypomagnesemia (serum magnesium <0.7 mmol/L), Yes (*n*, %)	13 (59.1)	44 (74.6)	0.18

**Table 4:** Detailed therapeutic intervention data for both treatment and control groups.

a. Treatment group data			
Variable	Number (%) or Median (IQR)		
Duration [days; median (IQR)]	3.9 (2.9–5.6)		
Initial dose [mg/kg/h] [median (IQR)]	0.1 (0.1–0.2)		
Number of patients requiring dose increase, Yes (*n*, %)	10 (45.5)		
Received additional therapy [Octreotide], Yes (*n*, %)	15 (68.2)		
Received additional therapy [Ranitidine], Yes (*n*, %)	1 (4.5)		
b. Control group data			
Variable	Number (%) or Median (IQR)		
	Intermittent omeprazole	Octreotide	Ranitidine
Number of patients (*n*)	52	10	6
Duration [days; median (IQR)]	10.4 (3.6–38.4)	5.2 (3.2–11.0)	2.6 (0.2–7.0)
Initial dose [median (IQR)]	0.87 (mg/kg/dose) (0.60–1.01)	1.00 (mcg/kg/h) (0.50–1.04)	0.98 (mg/kg/dose) (0.74–1.03)
Received additional therapy [Intermittent omeprazole], Yes (*n*, %)	NA	10 (100)	6 (100)
Received additional therapy [Octreotide], Yes (*n*, %)	10 (19.2)	NA	1 (16.7)
Received additional therapy [Ranitidine], Yes (*n*, %)	6 (11.5)	1 (10)	NA

### Additional outcomes and therapy data

The treatment group had a significantly lower incidence of all-cause mortality during admission compared to the control group (16 patients [72.7%] vs. 56 patients [94.9%], *p* = 0.005). The treatment group received continuous infusion of omeprazole, with a median (IQR) duration of 3.9 (2.9–5.6) days and a median (IQR) initial dose of 0.10 (0.08–0.17) mg/kg/h. A maximum dose of 0.38 mg/kg/h was reached with only one patient. Ten patients (45.5%) required a dose increase, and 68.2% and 4.5% of patients required the addition of octreotide and ranitidine, respectively. The control group received intermittent omeprazole (*n* = 52), with a median (IQR) duration of 10.4 (3.6–38.4) days and a median (IQR) initial dose of 0.87 (0.60–1.01) mg/kg/dose. Sixteen patients on intermittent omeprazole (30.8%) required additional therapy. In patients receiving octreotide (*n* = 10), the median (IQR) duration of therapy was 5.17 (3.2–11.04) days and a median (IQR) initial dose of 1 (0.50–1.04) mcg/kg/h. Lastly, patients receiving ranitidine (*n* = 6) had a median (IQR) duration of therapy of 2.62 (0.2–7) days and a median (IQR) initial dose of 0.98 (0.74–1.03) mg/kg/dose.

## Discussion

To our knowledge, this is the first study evaluating both the clinical benefits and safety of intravenous omeprazole continuous infusion in children with gastrointestinal bleeding. This gap in research makes it difficult to determine the most effective treatment options for this patient population (12). Our study revealed that the use of omeprazole IV continuous infusion was not associated with a reduction in the PICU LOS or the length of GI bleeding episode in comparison to other alternative intravenous therapies. However, the use of omeprazole IV continuous infusion showed lower rebleeding episodes and all-cause mortality compared to other intravenous therapies.

Although we could not find similar studies in pediatrics, limited data have evaluated the use of omeprazole IV continuous infusion in the adult population (9, 13, 14). In a prospective study of adult patients with active peptic ulcer bleeding or non-bleeding visible vessel who received either intermittent or continuous intravenous pantoprazole treatment, the mean duration of hospital stay was similar in both groups. In our study, we assessed PICU LOS which was longer in the treatment group (13). This can possibly be attributed to the severity of the critical condition of the patients included in our treatment group who had higher baseline rates of both upper and lower GI bleeding, coagulopathy, and liver cirrhosis. King Faisal Specialist Hospital and Research Centre (KFSHRC) is recognized as one of the few quaternary referral hospitals in the region, offering specialized and advanced healthcare services for a broad spectrum of pediatric conditions, including but not limited to oncology, medical genetics, cardiology, and gastroenterology. These populations with complex and advanced health conditions often experience severe and critical illnesses demanding intensive medical interventions and ongoing care. The intricate nature of their conditions poses challenges in identifying effective therapeutic approaches. Despite advancements in medical technology, these populations encounter significant challenges in achieving favorable health outcomes, which may result in increased mortality rates. In our study, we observed that continuous infusion of omeprazole was associated with a non-significant difference in rebleeding rates, which was slightly in favor of the treatment group. Our study also evaluated additional outcomes including all-cause mortality during hospital admission. All-cause mortality during hospital admission was significantly higher in the control group (94.9%). Such finding contrasts with a previous study involving adult patients with active non-variceal upper GI bleeding receiving either intermittent or continuous PPI infusion, where it was found that the mortality rate was, in fact, higher in patients receiving continuous PPI regimen mortality rate (1.7% vs. 7.3%, respectively, *p* = 0.308); however, the difference was not statistically significant (9). As mentioned earlier, our study was conducted on pediatric patients who were critically ill and admitted to the PICU, which could be one possible explanation for the discrepancy noted between the two studies.

In this study, we also assessed other secondary outcomes including rebleeding within 48 h from the resolution of the previous bleeding episode, transfusion requirements, and safety of therapy. No statistically significant differences were found between the treatment and control group in terms of rebleeding episodes, nausea and vomiting, and hypomagnesemia. Leung et al, conducted a retrospective multicenter review of adult patients with upper gastrointestinal bleeding to evaluate the rate of rebleeding after 48 h of endoscopy in patients receiving either continuous or intermittent PPI therapy. In the aforementioned study, rebleeding was defined as receiving ≥1 unit of blood product or requiring additional endoscopic, radiological, or surgical interventions. Their results showed that patients who received PPI continuous infusion had a higher rate of rebleeding (33.8% vs. 23.0%, *p* = 0.012); however, no difference was detected in the multivariable analysis which showed an adjusted odds ratio of 1.5 (95% confidence interval, 0.9–2.5) (14). While bacterial infections are a common concern in previous retrospective studies involving PPIs (15), our analysis of the study population revealed that none of the patients from the treatment group had an enteric infection. It is important to note that the incidence of bacterial infections can vary depending on factors such as geographic location, patient population, and study design. Regardless, our findings suggest that the use of continuous infusion of omeprazole does not pose an increased risk of developing enteric infections among patients with similar characteristics to those in our study.

There are limited data available on the dosage recommendations of omeprazole IV continuous infusion in the pediatric population. A case report on a 3-month-old infant with upper GI bleeding showed that the use of continuous intravenous infusion of omeprazole at a dose of 0.15 mg/kg/h contributed to controlling the patient's bleeding. However, rebleeding occurred after stopping the infusion, and omeprazole infusion was resumed with a higher dose of 0.3 mg/kg/h along with surgery until bleeding was controlled (6). In our study, the median (IQR) of the initial dose of omeprazole IV continuous infusion was 0.10 mg/kg/h (0.08–0.17) with a median (IQR) treatment duration of 3.9 days (2.9–5.6). Ten patients (45.5%) required dose escalation during their bleeding episodes. Our study showed a lower dose range than the case report above. Further studies are required to derive solid recommendations on optimal omeprazole IV continuous infusion dosing regimen for pediatric patients with GI bleeding. Lau et al., conducted a study to evaluate the efficacy of high-dose intermittent omeprazole in the prevention of recurrent bleeding compared with the placebo group in adult patients after endoscopic treatment. The study aimed to enroll 320 patients, with four interim analyses planned to assess rebleeding within 30 days. However, after enrolling only 240 patients, the study was terminated due to the significant difference (*p* < 0.001) found in favor of high-dose intermittent omeprazole (16). The importance of PPI therapy should not be undervalued due to a lack of data impacting patient outcomes, including mortality, surgery, or recurrent hemorrhage in the pediatric population. High-dose intermittent PPI therapy has been recommended to any patient presenting with significant upper GI bleeding until the cause is identified and treated (15, 17). Furthermore, in the pediatric population the use of omeprazole for the treatment of GI bleeding has been advised to control the bleeding and prevent rebleeding episodes (12).

Our study was based on a retrospective chart review design that had inherited limitations. Firstly, the nature of the study makes it difficult to generalize the results to a wider population. The interpretation of the data presented in Table 2 warrants caution due to potential influences from unaccounted factors such as replacements and transfusion procedures. These variables, which were not explicitly considered in our analysis, have the potential to impact the related parameters. In addition, the diagnostic procedures (e.g., endoscopy) and the severity of GI bleeding between the two groups were challenging to collect and assess for all patients. Therefore, careful consideration and further investigation are necessary to accurately interpret the findings derived from this dataset. Secondly, the sample size was small and large number of patients were excluded. This could have impacted the accuracy of the findings. However, the broad nature of our screening criteria which included retrieving data of all pediatric patients on omeprazole regardless of the route and administration may explain this reduction of patients included in our study. Thirdly, the study was conducted at a single center, which may have limited the range of data obtained. Finally, the variability among healthcare providers in dosing omeprazole continuous infusion and selection of therapies was at discretion of the treating physicians which may have affected the consistency and replicability of the results. Despite these limitations, our study provides valuable insights into the dosing of omeprazole continuous infusion in children with GI bleeding in a critical care setting, and monitoring parameters and highlights areas where further research is required. The empirical use of omeprazole continuous intravenous infusion for managing children with GI bleeding was less favorable when compared to alternative therapies in terms of shortening PICU LOS and duration of GI bleeding. However, our study results provide evidence supporting the safety and tolerability of empirical omeprazole continuous infusion use, suggesting it as a potential approach for managing GI bleeding in critically ill pediatric patients. Endoscopy is advisable to evaluate the actual cause of GI bleeding. Additional larger studies are necessary to determine the implication of omeprazole continuous infusion in this specific population.

## Data Availability

The raw data supporting the conclusions of this article will be made available by the authors, without undue reservation.
